# Multidrug-Resistant Tuberculosis in Child Successfully Treated with 9-Month Drug Regimen

**DOI:** 10.3201/eid2111.151119

**Published:** 2015-11

**Authors:** Jay Achar, Catherine Berry, Krzysztof Herboczek, Nargiza Parpieva, Mirzagaleb N. Tillyashaykhov, Zinaida N. Tigay, Atadjan Khamraev, Kalyan Velivela, James A. Seddon, Philipp du Cros

**Affiliations:** Médecins Sans Frontières, London, UK (J. Achar, K. Herboczek, J.A. Seddon, P. du Cros);; Médecins Sans Frontières, Tashkent, Uzbekistan (C. Berry, K. Velivela);; Ministry of Health, Tashkent (N. Parpieva, M.N. Tillyashaykhov);; Ministry of Health, Nukus, Uzbekistan (Z.N. Tigay, A. Khamraev);; Imperial College London, London (J.A. Seddon)

**Keywords:** multidrug-resistant, pediatric, treatment, bacteria, TB, tuberculosis, MDR-TB, Mycobacterium tuberculosis, antimicrobial resistance, children, Uzbekistan, paucibacillary TB

**To the Editor:** Approximately 480,000 persons acquired multidrug-resistant tuberculosis (MDR TB) in 2013 (*1*). Of the 32,000 children who acquire MDR TB annually, few are identified and administered appropriate treatment (*2*). World Health Organization (WHO)–recommended treatment for MDR TB lasts 20–24 months, including 8 months of daily drug injections (*3*). Because many children have paucibacillary TB, a shorter protocol may be sufficient, especially for early or nonsevere disease.

In Bangladesh, 87% of MDR TB patients who received a 9-month regimen had a favorable outcome; compared with patients who received the WHO regimen, fewer had adverse events or were lost to follow-up (*4*,*5*). A randomized controlled trial of this regimen is ongoing (*6*); however, the trial excludes children, and no detailed data are available for use of this regimen in children. The regimen is being implemented in several countries under operational research conditions (*7*).

Along with the Ministry of Health of Uzbekistan, and in accordance with WHO advice (*8*), Médecins Sans Frontières investigated the efficacy, tolerability, and safety of the shortened regimen in Karakalpakstan, Uzbekistan (*9*), where rates of second-line drug resistance are high (*1*) and *katG*-mediated isoniazid resistance predominates. Unlike the Bangladesh study (*4*), the Karakalpakstan study used moxifloxacin instead of gatifloxacin and included scheduled electrocardiograms (ECGs) and graded assessments of side effects to monitor for safety, including cardiac toxicity. All drugs in the regimen were previously used safely in children (*10*). For children, limited data are available regarding use of 2 new TB drugs, bedaquiline and delamanid; thus, the shortened regimen could represent the best opportunity to improve their outcomes and access to treatment. We report the successful treatment of MDR TB in a child who received the 9-month drug regimen. This retrospective research fulfilled Médecins Sans Frontières Ethics Review Board criteria for analysis of existing program data. Written informed consent was provided by the child and his parents.

In November 2013, a 14-year-old boy in Karakalpakstan received a diagnosis of pulmonary MDR TB after seeking medical care for a sore throat without cough, fever, weight loss, or major concurrent conditions. His mother (a close contact) had experienced symptoms of pulmonary TB since 2011 and, after a period of self-treatment, received a diagnosis of MDR TB with confirmed absence of preextensively or extensively drug-resistant TB; she completed appropriate treatment in September 2013. In accordance with national guidelines, the boy did not receive treatment for latent TB.

Clinical examination of the boy (weight 43 kg, body mass index 17.2 kg/m^2^) was unremarkable and showed no signs of extrapulmonary disease. A chest radiograph showed a left midzone interstitial infiltrate. Sputum sample testing (Xpert MTB/RIF; Cepheid, Sunnyvale, CA, USA) confirmed rifampin-resistant *Mycobacterium tuberculosis*. Sputum smear microscopy and liquid-based culture (BACTEC MGIT 960; Becton Dickinson, Franklin Lakes, NJ, USA) were negative. Baseline biochemical, hematologic, and ECG results were within normal limits. Serologic test results were negative for HIV and hepatitis B and C viruses.

Together, a history consistent with TB disease, radiographic evidence, and molecular testing results were considered sufficient indication for treatment of MDR TB. After psychosocial counseling and health education sessions, the boy, with his family’s agreement, consented to daily outpatient treatment with isoniazid (400 mg), ethambutol (800 mg), pyrazinamide (1,600 mg), prothionamide (500 mg), moxifloxacin (400 mg), capreomycin (750 mg), and clofazimine (100 mg) beginning in December 2013.

Treatment initiation was complicated by drug-associated nausea and vomiting, headache, tinnitus, and abdominal pain. Despite early aggressive management in line with study protocols, occasional vomiting continued. Intensive counseling ensured good adherence; only 3 days were missed. Corrected QT prolongation was excluded by use of ECG monitoring during treatment initiation.

After 4 months of treatment, the boy’s sputum smear microscopy and culture results remained negative, so the continuation phase of treatment was initiated. The daily regimen consisted of ethambutol (800 mg), pyrazinamide (1,600 mg), prothionamide (500 mg), moxifloxacin (400 mg), and clofazimine (100 mg).

In May 2014, after 6 months of treatment, the boy returned to school while still receiving treatment. In August 2014, the regimen was completed without incident, and at a 6-month follow-up, the boy had not experienced a relapse. According to study protocol, he will be followed for 1 year posttreatment to monitor for relapse.

The shortened treatment regimen has several potential benefits for children. The shorter treatment period enables an earlier return to school and social activities, the shorter duration of anti-TB injectable drug use may lessen ototoxicity, and fewer adverse effects and shorter duration could improve treatment adherence.

The reluctance to include children in TB research studies may result from difficulties in confirming a diagnosis (due to paucibacillary disease and difficulty in obtaining specimens); such confirmation is often a prerequisite for treatment. Other barriers include lack of second-line TB drug formulations and pharmacokinetic data for children, ethics review issues, and informed and parental consent issues. Clinicians and TB program managers could consider the 9-month treatment regimen for children. We advocate inclusion of children of all ages in research investigating the efficacy and safety of a 9-month regimen and emphasize the importance of separately reporting data for children.
